# Burden and Socio-Behavioral Correlates of Uncontrolled Abnormal Glucose Metabolism in an Urban Population of India

**DOI:** 10.1371/journal.pone.0163891

**Published:** 2016-10-18

**Authors:** Tanmay Mahapatra, Kaushik Chakraborty, Sanchita Mahapatra, Umakanta Mahapatra, Naren Pandey, Peter L. Thomson, Arthur W. Musk, Ramendra N. Mitra

**Affiliations:** 1 Barrackpore Population Health Research Foundation, Kolkata, 700123, India; 2 Mission Arogya Health and Information Technology Research Foundation, Kolkata, 700010, India; 3 Department of Endocrinology and Metabolism, Institute of Post-Graduate Medical, Education and Research, Kolkata, 700020, India; 4 Mediland Diagnostic Institute, Kolkata, Kolkata, 700014, India; 5 Sir Charles Gairdner Hospital, University of Western Australia, Nedlands, 6009, Australia; 6 School of Population Health, University of Western Australia, Perth, 6009, Australia; McGill University, CANADA

## Abstract

**Background:**

Progressive burden of diabetes mellitus is a major concern in India. Data on the predictors of poor glycemic control among diabetics are scanty. A population-based cross-sectional study nested in an urban cohort was thus conducted in West Bengal, India to determine the burden and correlates of total and uncontrolled abnormalities in glucose metabolism (AGM) in a representative population.

**Methods:**

From 9046 adult cohort-members, 269 randomly selected consenting subjects (non-response = 7.24%) were interviewed, examined [blood pressure (BP), anthropometry], tested for fasting plasma glucose (FPG) and glycosylated hemoglobin (HbA1C). Those having pre-diagnosed diabetes or FPG ≥126 or HbA1c≥6.5 were defined as diabetic. Among non-diabetics, subjects with FPG (mg/dl) = 100–125 or HbA1C(%) = 5.7–6.4 were defined as pre-diabetic. Pre-diagnosed cases with current FPG ≥126 were defined as uncontrolled AGM. Descriptive and regression analyses were conducted using SAS-9.3.2.

**Results:**

Among participants, 28.62% [95% Confidence Interval (95%CI) = 23.19–34.06)] were overweight [body mass index(BMI) = (25–29.99)kg/meter^2^], 7.81% (4.58–11.03) were obese(BMI≥30kg/meter^2^), 20.82% (15.93–25.70) were current smokers, 12.64% (8.64–16.64) were current alcohol-drinkers and 46.32% of responders (39.16–53.47) had family history of diabetes. 17.84% (13.24–22.45) had stage-I [140≤average systolic BP (AvSBP in mm of mercury)<160 or 90≤average diastolic BP (AvDBP)<100] and 12.64% (8.64–16.64) had stage-II (AvSBP≥160 or AvDBP≥160) hypertension. Based on FPG and HbA1c, 10.41% (6.74–14.08) were diabetic and 27.88% (22.49–33.27) were pre-diabetic. Overall prevalence of diabetes was 15.61% (11.25–19.98). Among pre-diagnosed cases, 46.43% (26.74–66.12) had uncontrolled AGM. With one year increase in age [Odds Ratio(OR) = 1.05(1.03–1.07)], retired subjects [OR = 9.14(1.72–48.66)], overweight[OR = 2.78(1.37–5.64)], ex-drinkers [OR = 4.66(1.35–16.12)] and hypertensives [OR_Stage I_ = 3.75(1.42–9.94); OR_Stage II_ = 4.69(1.67–13.17)] had higher odds of diabetes. Relatively older subjects [OR = 1.06(1.02–1.10)], unemployed [OR = 19.68(18.64–20.78)], business-owners [OR = 25.53(24.91–16.18)], retired [OR = 46.53(45.38–47.72)], ex-smokers [OR = 4.75(1.09–20.78)], ex-drinkers [OR = 22.43(4.62–108.81)] and hypertensives [OR_Stage II_ = 13.17(1.29–134.03)] were more likely to have uncontrolled AGM.

**Conclusions:**

Burden of uncontrolled AGM was high among participants. Efforts to curb the diabetes epidemic in urban India should include interventions targeting appropriate diabetic control among relatively older persons, unemployed, business-owners, retired, ex-smokers, ex-drinkers and hypertensives.

## Introduction

Diabetes Mellitus is a heterogeneous syndrome of abnormalities in carbohydrate and fat metabolism, characterized by multi-factorial interplay between genetic and environmental factors culminating into beta-cell dysfunction and reduced tissue insulin sensitivity.[1] Most adult type 2 diabetics are found to be overweight and centrally obese. The three explanatory paradigms are: portal/visceral hypothesis (delineating the key role of elevated non-esterified fatty acids), ectopic fat storage (triglyceride deposits in muscle/liver/pancreas) and role of adipose tissue endocrine organs (secretions of various adipocytokines implicated in insulin resistance and probably beta-cell dysfunction).[2–4] Thus an obesogenic environment comprising of lifestyle, eating habits, addictions (smoking and alcoholism), stress and physical inactivity, are considered to be the predominant yet modifiable determinants of diabetes.[5] On the other hand in case of Type 1 diabetes (immune-mediated, non-autoimmune or Idiopathic leading to absolute insulin deficiency) of younger age group,the hyperglycemia is due to Beta cell destruction mostly with immunologic marker from insulin deficiency.

Diabetes has become a major public health challenge due to its increasing prevalence in most countries.[6,7] The International Diabetes Federation (IDF) estimated that in 2012, globally 4.8 million persons died of diabetes while 347 million were suffering from it (adult prevalence of 8.3%).[6] More than 80% of these cases and deaths were reported from low and middle income countries, while India and China contributed the most.[6,8] The burden of diabetes was reported to have increased rapidly in India.[9,10] The IDF estimated the prevalence of diabetes in India to be 9.01% among persons aged ≥20 years in 2012.[6,11] According to the India Diabetes (ICMR–INDIAB) study by ICMR in 2011, approximately 62.4 million people aged ≥20 years were suffering from diabetes and the finding was consistent with that of IDF.[6,11]

Reviews on abnormalities in glucose metabolism (AGM) in India showed that the majority of the earlier studies were conducted in southern and northern parts of the country [12–14] and a few in the northeastern states. Data from the eastern region, particularly from the populous state of West Bengal, are scanty [12–15]. Related studies were mostly concerned with prevalence rather than risk factors, the predictors of uncontrolled disease, or the population subgroups to target preventive and treatment efforts.[16,17] Moreover, methodological shortcomings related to study design, sampling, geographical diversities, non-standardized diagnostic criteria, incomplete data, high non-response and non-representative sampling precluding extrapolation of observations beyond the study samples.[12]

A dearth of information on socio-behavioral determinants of uncontrolled AGM thus called for a detailed investigation in a representative, population-based sample. A cross-sectional study nested in a cohort of an urban population in West Bengal was thus conducted with following objectives:

1. To estimate the prevalence of overall and uncontrolled cases of AGM among adults and
2. To measure the strength of association between these abnormalities and their socio-demographic, physical and behavioral correlates.

## Methods

### Study population

An open population cohort was established in 2001 by Barrackpore Population Health Research Foundation in North 24 Parganas district of West Bengal to identify risk factors of non-communicable diseases, to measure their burden and to control them through appropriate interventions. From 24 administrative divisions (termed “Wards”) in Barrackpore (having 144391 persons residing in 31715 household as per the 2001census), six were chosen randomly (Ward No. 3, 6, 11, 15, 18 and 22) where,all households were identified and enumerated. Using empirical most conservative parameter value of 50%, assuming α = 0.05, as per the sample size calculation formula: s = z^2^ *p(1-p)/d^2^, (d = desired precision = 5% of 50 = 2.5) and finite population correction formula: n = s/(1 + (s/N)) where N = population size = 31715 households altogether, 489 (using Epi Info) households from each of the six randomly selected wards were required to be recruited assuming equal cluster (ward) population size and empirically assumed designed effect = 2.

Hence from the list of all houses in each of the six selected wards approximately 500 households were selected randomly by using random number generation and selection using computer programming. The number of selected household in each selected ward did not remain exactly 500 because some of the randomly selected identification numbers for one household actually represented more than one because there was more than one family residing (and cooking separately) at the same address. Thus 3030 households from six selected wards with 12734 residents were included in the cohort at baseline in 2001 and are being continuously followed. People were removed from the cohort by death or migration-out and were included by birth or migration-in.

### Sample size for current study and recruitment

Due to non-availability of appropriate parameter values, the previously observed burden of abnormal FPG levels within this cohort (in 2006 among 1194 randomly selected representative individuals 22.65% had FPG≥100mg/dl) was used for the sample size calculation.[18,19]

**Fig 1:**
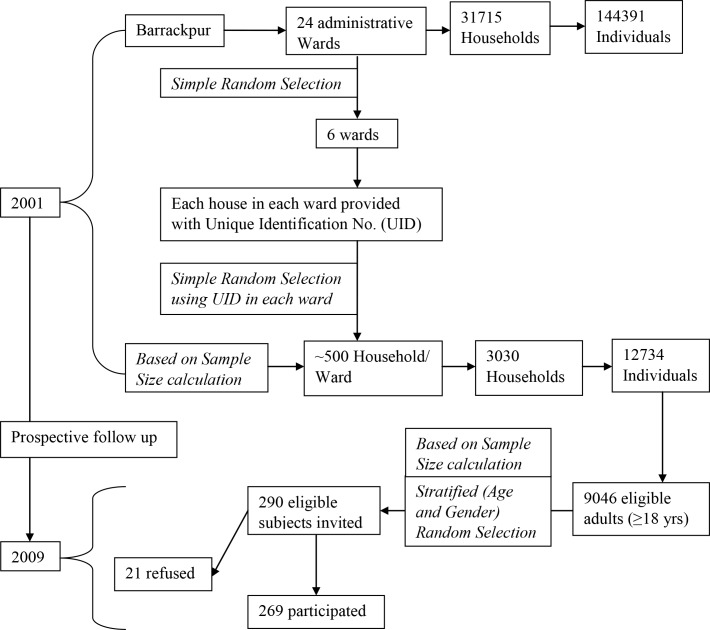
Flowchart the recruitment process of the original cohort and the current study nested within it.

Based on this parameter estimate in the source population, using the population-survey tool of Epi-Info,[20] to determine the prevalence of abnormal FPG level among adults in the study population with a relative precision of 10% and ∞ = 0.05, 261 subjects were required for a cross-sectional study. Exclusion criteria included: migration out, refusal to provide informed consent and non-availability in three consecutive follow-ups. Assuming a non-response rate of 10%, 290 adults (irrespective of their diabetic and treatment status) were randomly selected from the list of all 9046 adult cohort members in 2009 and invited to participate between November 2009 and March 2010 (using age and gender stratification to preserve the distribution). Altogether 269 of these 290 invited eligible subjects provided consent, were interviewed, tested and included in the analysis. (Fig 1)

### Ethics statement

Prior to the interview and blood sample collection, written informed consent was collected. Data were securely preserved with confidentiality. The study protocol was reviewed and approved by the Institutional Ethics Committee of National Institute of Cholera and Enteric Diseases Kolkata (Reference no.: C-48/2010 T & E) and Barrackpore Population Health Research Foundation, Barrackpore (Reference no.: BKPHS-RKVM/April, ‘09/03).

### Interview and measurements

Using a pre-tested, structured questionnaire, information was collected on socio-demographics, anthropometry, personal behaviors and habits along with familial/personal history of diagnosed diabetes. Socio-demographic information included: age, gender (male/female) and occupation (laborer/service/never worked (student/unemployed/housewife)/self-employed or in business/retired). Measured body-mass index (kg/meter^2^) was categorized into underweight (<18.5), normal (18.5–24.99), overweight (25–29.99) and obese (30 or more).[21,22] Using anatomical landmarks (at the midpoint between the lower rib and iliac crest) waist circumference (cm) was measured [23–25] to determine abdominal obesity categorized as: no (≤90 for men and ≤80 for women) and yes (>90 for men and >80 for women).[26] All anthropometric measurements were conducted by trained personnel, specifically recruited for this purpose.

Personal behavior/habits included physical exercise (hour/wk), smoking and alcohol-drinking. For the quantification of smoking, number of cigarettes smoked/day (converted to packs smoked/day assuming Indian standard pack of 10 cigarettes), year of starting smoking and duration of smoking were determined. Pack-years (py) were calculated by combining the frequency and duration. Type of smoking was then categorized into non-smoker/ex-smoker/current light smoker (0.1–20.0py)/current moderate smoker (20.1–40.0py)/current heavy smoker (>40py).[27] Based on similar questions, type of alcohol-drinking was classified as: non-drinker/ex-drinker/infrequent drinker (drinking rarely or 1–3 times/month)/frequent drinker (once wkly ≤ drinking <daily)/daily drinker. Duration of alcohol intake in completed years, family history of diabetes (yes/no/don’t know) and whether the subject had already been diagnosed as a diabetic (yes/no) were enquired additionally.[28]

Three arterial blood pressure readings were taken at 10 minutes interval with a bound of ±3 mm of Hg and the averages (mm of Mercury) were used to define hypertension status as: normotensive [average systolic BP (AvSBP)<120 and average diastolic BP (AvDBP)<80), pre-hypertensive [(120≤AvSBP<140 and AvDBP<90) or (AvSBP<140 and 80≤AvDBP<90)], stage-I hypertension [(140≤AvSBP<160 and AvDBP<100) or (AvSBP<160 and 90≤AvDBP<100)] and stage-II hypertension (AvSBP≥160 or AvDBP ≥100).[29]

### Laboratory testing and parameters

To determine AGM through several parameters (observed level of FPG, glycemic control, current diagnosis of diabetes and overall diagnosis) and then to define AGM status based on demonstrated control over the aforementioned parameters we needed to measure FPG and glycosylated hemoglobin (HbA1c). For this purpose, approximately 3ml of venous blood was collected in double oxalate and EDTA vials on the day of interview, from each participant after overnight fasting. Plasma separation from the double-oxalated blood was done in the study laboratory in Barrackpore and then carried in a cold chamber to the central laboratory at National Institute of Cholera and Enteric Diseases, Kolkata where preparation for assays and storage were performed. Samples were next sent to the processing Laboratory at Mediland, Diagnostic Institute, Kolkata in duly labeled Eppendorf tube maintaining a temperature of 2–8°C. For HbA1cwhole blood in EDTA vials was carried to the same laboratory in a cool box maintaining a temperature of 2–8°C. Samples were stored for the next six months in a refrigerator at -20 degree centigrade temperature.

FPG was estimated from plasma separated from the double oxalated blood using GOD/POD enzymatic method on fully automated Biochemistry analyzer [A-25 Biosystems (Spain)] and the assay took 5 minutes time/sample. A. HbA1c was estimated from whole blood collected in EDTA vials, using Column Method [Biosystems (Spain)] taking 30 minutes /sample.

Initially three outcome parameters regarding AGM were defined and categorized as follows:

1. Observed FPG level in mg/dl into 1. Normal (if FPG <100); 2. Impaired (FPG = 100–125) or 3. Diabetic (FPG≥126).[18,19]
2. Observed HbA1c level (%) into: having 1. Normal (<5.7), 2. Pre-diabetic (5.7–6.4) or Diabetic (≥6.5) level of glycemic control.[18,19]
3. Current diagnosis of diabetes based on both observed FPG and HbA1C together into 1. Non-diabetic (FPG<100 mg/dl and HbA1c<5.7%), 2. Pre-diabetic [(FPG<100 and 5.7≤ HbA1c <6.5) or (100≤ FPG <126 and HbA1c <5.7)] or 3. Diabetic (FPG≥126 mg/dl or HbA1c≥6.5) level of biochemical picture.[18,19]

Then combining the answer to the question about ever being diagnosed before with diabetes (also substantiated by checking medical records available at the time of interview) with the outcome parameter: Current diagnosis of diabetes, the next parameter for AGM: Overall diagnosis (based on previous and current diagnosis) was defined and classified into 1. Diabetic: those who had been previously diagnosed as diabetic and/or had diabetic level of current biochemical picture (FPG≥126 mg/dl or HbA1c≥6.5) or 2. Non-diabetic.

Next, among previously diagnosed diabetics, AGM status (based on prior diagnosis of diabetes: yes/no and current FPG results) was categorized as: 1. Never diagnosed with AGM (non-diabetic FPG with no prior diagnosis of diabetes), 2. Well-controlled AGM (non-diabetic FPG with prior diagnosis of diabetes) 3. Moderately-controlled AGM (pre-diabetic FPG with prior diagnosis of diabetes) or 4. Un-controlled AGM (diabetic FPG with prior diagnosis of diabetes).[30]

### Data analysis

Following descriptive analyses [means with standard deviation (SD) and proportions with 95% Confidence Intervals (95%CI)], measurement of the strengths of associations [Odds Ratios (OR) and corresponding 95%CIs] of potential socio-demographic, physical and behavioral correlates with binary (prior diagnosis of diabetes, type of diagnosis of abnormal FPG and glycemic control) and multi-category (FPG level, glycemic control and AGM status) outcome variables were conducted using simple and multinomial bivariate logistic regressions respectively,[31–33] using SAS version 9.3.2.[34]

We modeled fasting plasma glucose level (ref: normal), glycemic control (reference: normal), current diagnosis of diabetes (reference: non-diabetic), overall diagnosis of diabetes (reference: non-diabetic) and AGM status as our five outcome variables and regressed each of them on age, gender (reference = female), occupation (reference = laborer), physical exercise (hrs/wk), body mass index (reference = normal), waist circumference (cm), abdominal obesity (reference = no), smoking category (reference = non-smoker), completed years of alcohol drinking, type of alcohol drinking (reference = non-drinker), family history of diabetes (reference = no) and diagnosis of hypertension (reference = normo-tensive) separately.

## Results

### Socio-demographics and anthropometric measures

Among 269 randomly selected subjects (134 males and 135 females), mean age was 42.49 (SD = 16.29) years, 49.81% (43.80–55.83) never worked, 8.18% (4.88–11.47) were retired while only 14.50% (10.26–18.73) were self-employed or in business.

Mean BMI was 23.53 kg/m^2^, 28.62% (23.19–34.06) were overweight and 7.81% (4.58–11.03) were obese. Abdominal obesity was present in 37.55% (31.72–43.37). (Table 1)

**Table 1:** Distribution of socio-behavioral and physical characteristics, fasting plasma glucose (FPG), glycemic control, diabetes/AGM and hypertension among 269 randomly selected subjects in an urban population of India.

Variables		Male (N = 134)		Female (N = 134)		Total	
Continuous		Mean	SD	Mean	SD	Mean	SD
Age in years		42.82	16.81	42.17	15.81	42.49	16.29
Physical exercise (hrs/wk)		5.56	17.16	3.74	14.55	4.65	15.90
Pack-years of smoking		8.29	18.36	0.00	0.00	4.13	13.59
Duration of alcohol intake in years		5.69	11.41	0.00	0.00	2.84	8.53
Body Mass Index (kg/squared meter)		22.97	3.98	24.08	4.88	23.53	4.48
Waist circumference (cm)		83.80	10.67	80.33	13.03	82.06	12.02
Fasting blood glucose		97.28	28.56	94.84	23.50	96.06	26.12
Glycosylated haemoglobin		5.61	0.68	5.56	0.60	5.59	0.64
**Categorical Variables**	**Categories**	**N**	**%**	**N**	**%**	**N**	**%**
Gender	Male					134	49.81(43.80–55.83)
	Female					135	50.19(44.17–56.20)
Occupation	Never worked	17	12.67	118	87.41	134	49.81(43.80–55.83)
	Laborer	25	18.66	9	6.67	34	12.64(8.64–16.64)
	Service	35	26.12	5	3.70	40	14.87(10.59–19.15)
	Self-employed/Business	36	26.87	3	2.22	39	14.50(10.26–18.73)
	Retired	21	15.67	1	0.74	22	8.18(4.88–11.47)
Body Mass Index	Underweight	18	13.43	18	13.33	36	13.38(9.29–17.48)
	Normal	75	55.97	60	44.44	135	50.19(44.17–56.20)
	Overweight	35	26.12	42	31.11	77	28.62(23.19–34.06)
	Obese	6	4.48	15	11.11	21	7.81(4.58–11.03)
Abdominal obesity	No	100	74.63	68	50.37	168	62.45(56.63–68.28)
	Yes	34	25.37	67	49.63	101	37.55(31.72–43.37)
Smoking category	Non-smoker	58	43.28	135	100.00	193	71.75(66.33–77.16)
	Ex-smoker	20	14.93	0	0.00	20	7.43(4.28–10.59)
	Current light smoker	39	29.10	0	0.00	39	14.50(10.26–18.73)
	Current moderate smoker	5	3.73	0	0.00	5	1.86(0.23–3.48)
	Current heavy smoker	12	8.96	0	0.00	12	4.46(1.98–6.94)
Type of alcohol drinking	Non-drinker	89	66.42	135	100.00	224	83.27(78.78–87.76)
	Ex-drinker	11	8.21	0	0.00	11	4.09(1.71–6.47)
	Current drinker	34	25.37	0	0.00	34	12.64(8.64–16.64)
Family history of diabetes	Yes	52	59.77	50	48.54	102	53.68(46.53–60.84)
	No	35	40.23	53	51.45	88	46.32(39.16–53.47)
Hypertension status	Normotensive	46	34.33	52	38.52	98	36.43(30.64–42.22)
	Pre-hypertensive	43	32.09	46	34.07	89	33.09(27.43–38.74)
	Stage I Hypertension	27	20.15	21	15.56	48	17.84(13.24–22.45)
	Stage II Hypertension	18	13.43	16	11.85	34	12.64(8.64–16.64)
Previously diagnosed as a diabetic	No	115	85.82	126	93.33	241	89.59(85.92–93.26)
	Yes	19	14.18	9	6.67	28	10.41(6.74–14.08)
Fasting plasma glucose (FPG) level	Normal	97	72.39	97	71.85	194	72.12(66.73–77.51)
	Impaired	25	18.66	29	21.48	54	20.07(15.26–24.89)
	Diabetic	12	8.96	9	6.67	21	7.81(4.58–11.03)
Glycemic control	Normal	85	63.43	88	65.19	173	64.31(58.55–70.07)
	Pre-diabetic	35	26.12	35	25.93	70	26.02(20.75–31.30)
	Diabetic	14	10.45	12	8.89	26	9.67(6.11–13.22)
Current diagnosis regarding Diabetes (based on both FPG and HbA1c)	Non-diabetic	84	62.67	82	60.74	166	61.71(55.86–67.56)
	Pre-diabetic	36	26.87	39	28.89	75	27.88(22.49–33.27)
	Diabetic	14	10.45	14	10.37	28	10.41(6.74–14.08)
Overall diagnosis (based on previous and current diagnosis)	Non-diabetic	112	83.58	115	85.19	227	84.39(80.02–88.75)
	Diabetic	22	16.42	20	14.81	42	15.61(11.25–19.98)
AGM status (based on prior diagnosis and current FPG)	Well-controlled	2	10.53	4	44.44	6	21.43(5.23–37.63)
	Moderately-controlled	7	36.84	2	22.22	9	32.14(13.70–50.58)
	Un-controlled	10	52.63	3	33.33	13	46.43(26.74–66.12)

N = Number of subjects, SD = Standard deviation, AGM = abnormalities in glucose metabolism

### Behavioral factors and family history

Subjects reported an average of 4.65 hours of physical exercise/week. There were no female smoker or drinker among the participants which appeared a bit unusual. Overall there were 20.82% current smokers [including 4.46% (1.98–6.94) heavy smokers], 7.43% (4.28–10.59) ex-smokers, 12.64% (8.64–16.64) current alcohol-drinkers and 4.09% (1.71–6.47) ex-drinkers. Among those who could remember, 46.32% (39.16–53.47) had family history of diabetes. (Table 1)

### Hypertension

The proportion of stage-I and II hypertensive residents were 17.84% (13.24–22.45) and 12.64% (8.64–16.64) respectively. (Table 1)

### AGM

Prior to this study 10.41% (6.74–14.08) subjects were diagnosed as diabetics. In our study 20.07% (15.26–24.89) had impaired FPG while 7.81% (4.58–11.03) had that of diabetic level. According to HbA1c results, 26.02% (20.75–31.30) and 9.67% (6.11–13.22) had pre-diabetic and diabetic level of glycemic control respectively. (Table 1)

Based on both tests, currently10.41% (6.74–14.08) of subjects had diabetic and 27.88% (22.49–33.27) had pre-diabetic levels of glucose metabolism. Overall (including prior and current diagnosis) the prevalence of diabetes was 15.61% (11.25–19.98). Among previously diagnosed diabetics, 46.43% (26.74–66.12) had uncontrolled AGM. (Table 1)

### Association of socio-demographics and anthropometric measures with AGM

One year increase in age was associated with higher odds of having impaired (OR = 1.03, 95%CI = 1.01–1.05) and diabetic (OR = 1.05, 95%CI = 1.02–1.08) level of FPG, diabetic level of glycemic control (OR = 1.05, 95%CI = 1.03–1.08) and being diabetic (currently: OR = 1.05, 95%CI = 1.03–1.08 and overall OR = 1.05, 95%CI = 1.03–1.08). Retired (with reference to the laborer) residents were more likely to have diabetic level of FPG (OR = 10.77, 95% CI = 1.14–101.72) and HbA1c (OR = 10.50, 95%CI = 1.13–97.90) while they had higher odds of being diabetic (currently OR = 10.50, 95%CI = 1.13–97.90 and overall OR = 9.14, 95%CI = 1.71–48.66). The likelihood of having impaired FPG (OR = 3.01, 95%CI = 1.50–6.05), diabetic level of HbA1c (OR = 2.47, 95%CI = 1.03–5.87) and being diabetic (currently: OR = 2.53, 95%CI = 1.09–5.91; overall: OR = 2.78, 95%CI = 1.37–5.64) were higher among overweight subjects (reference: normal weight). Higher waist circumference was associated with increased likelihood of having impaired (OR = 1.03, 95%CI = 1.00–1.06) as well as diabetic (OR = 1.05, 95%CI = 1.01–1.09) level of FPG, diabetic level of HbA1c (OR = 1.05, 95%CI = 1.02–1.09) and having diabetes (currently: OR = 1.05, 95%CI = 1.02–1.09; overall: OR = 1.06, 95%CI = 1.03–1.09). (Table 2)

**Table 2:** Socio-behavioral and physical correlates and their association with abnormal fasting plasma glucose level, glycemic control and AGM among the participants (N = 269).

Variables	Categories	Fasting plasma glucose level (Ref: Normal)		Glycemic Control (Reference: Normal)		Current Diagnosis of Diabetes (Reference: Non-diabetic)		Overall Diagnosis of Diabetes (Reference: Non-diabetic)
		Impaired	Diabetic	Pre-diabetic	Diabetic	Pre-diabetic	Diabetic	
		OR(95% CI)	OR(95% CI)	OR(95% CI)	OR(95% CI)	OR(95% CI)	OR(95% CI)	OR(95% CI)
Age		**1.03(1.01–1.05)**	**1.05(1.02–1.08)**	1.01(0.99–1.03)	**1.05(1.03–1.08)**	1.01(0.99–1.03)	**1.05(1.03–1.08)**	**1.05(1.03–1.07)**
Gender (Reference = Female)	Male	0.86(0.47–1.57)	1.33(0.54–3.31)	1.04(0.59–1.80)	1.21(0.53–2.76)	0.90(0.52–1.56)	0.98(0.44–2.17)	1.13(0.58–2.18)
Occupation (Reference = Laborer)	Never worked	1.62(0.57–4.58)	2.60(0.32–21.39)	0.60(0.26–1.35)	2.77(0.34–22.49)	0.72(0.32–1.62)	3.46(0.43–27.80)	2.64(0.59–11.95)
	Service	1.27(0.36–4.44)	1.81(0.16–21.02)	0.71(0.26–1.93)	1.56(0.13–18.34)	0.81(0.30–2.16)	1.61(0.14–19.07)	3.39(0.66–17.58)
	Self-employed/Business	2.24(0.67–7.45)	4.48 (0.47–42.79)	0.96(0.35–2.60)	4.77(0.51–44.33)	0.96(0.35–2.59)	4.77(0.51–44.33)	2.90 (0.55–15.49)
	Retired	1.72(0.40–7.50)	**10.77(1.14–101.72)**	0.58(0.15–2.21)	**10.50(1.13–97.90)**	0.58(0.15–2.22)	**10.50(1.13–97.91)**	**9.14 (1.71–48.66)**
Physical exercise(hrs/wk)		1.01(0.99–1.03)	1.01(0.99–1.03)	0.98(0.95–1.01)	1.01(0.99–1.03)	0.99(0.97–1.01)	1.01(0.99–1.03)	1.02(0.99–1.03)
Body Mass Index (Reference = Normal)	Underweight	1.33(0.51–3.48)	-	1.09(0.48–2.52)	-	0.96(0.42–2.19)	-	0.20(0.03–1.54)
	Overweight	**3.01(1.50–6.05)**	1.95(0.76–5.04)	1.71(0.89–3.26)	**2.47(1.03–5.87)**	1.75(0.92–3.30)	**2.53(1.09–5.91)**	**2.78(1.37–5.64)**
	Obese	1.38(0.42–4.59)	0.60(0.07–4.94)	0.71(0.22–2.29)	0.47(0.06–3.90)	0.62(0.19–1.99)	0.42(0.05–3.43)	0.73(0.16–3.42)
Waist circumference (cm)		**1.03(1.00–1.06)**	**1.05(1.01–1.09)**	1.01(0.98–1.03)	**1.05(1.02–1.09)**	1.01(0.99–1.03)	**1.05(1.02–1.09)**	**1.06(1.03–1.09)**
Abdominal obesity (Reference = No)	Yes	1.67(0.91–3.08)	1.76(0.71–4.37)	1.49(0.84–2.63)	1.98(0.86–4.55)	1.50(0.86–2.63)	2.02(0.90–4.53)	**2.31(1.19–4.50)**
Smoking category (Reference = Non-smoker)	Ex-smoker	1.36(0.45–4.08)	2.45(0.62–9.72)	1.89(0.67–5.35)	3.06(0.85–10.98)	1.63(0.58–4.59)	2.60(0.73–9.26)	2.53(0.90–7.12)
	Current light smoker	**0.28(0.08–0.96)**	0.28(0.04–2.20)	0.55(0.23–1.33)	0.22(0.03–1.73)	0.47(0.20–1.14)	0.19(0.02–1.46)	0.32(0.07–1.40)
	Current moderate smoker	2.18(0.35–13.45)	-	1.62(0.26–9.99)	-	1.39(0.23–8.57)	-	1.47(0.16–13.67)
	Current heavy smoker	0.93(0.19–4.66)	4.19(0.97–18.06)	1.22(0.29–5.05)	3.44(0.79–15.00)	1.05(0.25–4.33)	2.93(0.68–12.66)	4.21(1.25–14.19)
Completed years of alcohol drinking		1.02(0.98–1.05)	**1.05(1.01–1.08)**	1.02(0.99–1.06)	**1.04(1.00–1.08)**	1.02(0.98–1.05)	1.04(0.99–1.07)	1.02(0.99–1.06)
Type of alcohol drinking (Reference = Non-drinker)	Ex-drinker	2.66(0.57–12.30)	**10.87(2.47–47.90)**	1.89(0.41–8.70)	**7.30(1.69–31.51)**	1.66(0.36–7.61)	**6.32(1.47–27.13)**	**4.66(1.35–16.12)**
	Current drinker	0.66(0.24–1.80)	0.81(0.17–3.72)	0.99(0.43–2.26)	0.64(0.14–2.90)	0.86(0.38–1.97)	0.55(0.12–2.50)	0.54(0.16–1.87)
Family history of diabetes (Reference = No)	Yes	**2.68(1.20–5.96)**	1.03(0.34–3.09)	1.09(0.55–2.14)	0.98(0.37–2.57)	1.21(0.63–2.35)	1.12(0.43–2.91)	1.79(0.78–4.08)
Diagnosis of hypertension (Reference = Normo-tensive)	Pre-hypertensive	1.99(0.94–4.23)	3.09(0.77–12.46)	1.88(0.98–3.59)	2.44(0.68–8.76)	1.49(0.79–2.81)	3.02(0.88–10.35)	1.75(0.68–4.51)
	Stage I Hypertension	1.81(0.73–4.49)	**5.06(1.19–21.45)**	0.82(0.33–2.05)	**5.92(1.72–20.35)**	0.66(0.27–1.63)	**5.58(1.62–19.23)**	**3.75(1.42–9.94)**
	Stage II Hypertension	2.60(0.99–6.87)	**6.75(1.49–30.61)**	1.18(0.46–3.01)	**4.22(1.04–17.16)**	1.31(0.54–3.17)	**4.41(1.08–18.06)**	**4.69(1.67–13.17)**

OR = Odds Ratio 95%CI = 95% confidence interval

Boldfaced figures indicate results for which p value was <0.05

“-”refers to situations when valid Odds Ratio could not be determined due to lack of adequate observations

### Association of behavioral factors and family history with AGM

Risk of having diabetic level of FPG (OR = 1.05, 95%CI = 1.01–1.08) and HbA1c (OR = 1.04, 95%CI = 1.00–1.08) increased with duration of alcohol drinking. Compared to non-drinkers, ex-drinkers had higher odds of having diabetic level of FPG (OR = 10.87, 95%CI = 2.47–47.9), HbA1c (OR = 7.30, 95%CI = 1.69–31.51) and having diabetes (currently: OR = 6.32, 95%CI = 1.47–27.13; overall: OR = 4.66, 95%CI = 1.35–16.12).

Subjects with (reference: no) family history of diabetes had higher (OR = 2.68, 95%CI = 1.20–5.96) likelihood of having impaired FPG. (Table 2)

### Association between Hypertension and AGM

With reference to normotensive subjects, hypertensives were more likely to have diabetic level of FPG (OR_Stage-I Hypertension_ = 5.06, 95%CI = 1.19–21.45; OR_Stage-II Hypertension_ = 6.75, 95%CI = 1.49–30.61) and HbA1c (OR_Stage-I Hypertension_ = 5.92, 95%CI = 1.72–20.35; OR_Stage-II Hypertension_ = 4.22, 95%CI = 1.04–17.16) as well as had higher likelihood of being diabetic (currently: OR_Stage-I Hypertension_ = 5.58, 95%CI = 1.62–19.23; OR_Stage-II Hypertension_ = 4.41, 95%CI = 1.08–18.06 and overall: OR_Stage-I Hypertension_ = 3.75, 95%CI = 1.42–9.94; OR_Stage-II Hypertension_ = 4.69, 95%CI = 1.67–13.17). (Table 2)

### Correlates of uncontrolled AGM

Control of AGM was evaluated with reference to never-diagnosed (those who were neither diagnosed previously nor during the study) owing to the small number of cases diagnosed earlier. One year increase in age increased the likelihood (OR = 1.06, 95%CI = 1.02–1.10) of having uncontrolled AGM. (Table 3)

**Table 3:** Association of socio-behavioral and physical characteristics with AGM status (Reference: Never diabetic) during the study (based on current fasting plasma glucose and past diagnosis of diabetes) among the participants (N = 269).

Variables	Categories	AGM status (Reference = Never diabetic)		
		Well-controlled	Moderately-controlled	Un-controlled
		OR(95% CI)	OR(95% CI)	OR(95% CI)
Age		1.05(1.00–1.10)	**1.06(1.01–1.10)**	**1.06(1.02–1.10)**
Gender (Reference = Female)	Male	0.49(0.09–2.74)	3.43(0.69–16.92)	3.26(0.87–12.23)
Occupation (Reference = Laborer)	Service	**43.00(41.94–44.09)**	**1.98(1.91–2.05)**	**9.40(9.07–9.74)**
	Business	**1.23(1.05–1.44)**	**1.27(1.21–1.34)**	**25.53(24.91–26.18)**
	Retired	**0.72(0.55–0.94)**	**3.70(3.56–3.85)**	**46.53(45.38–47.72)**
Body mass Index (Reference = Normal)	Underweight	-	1.18(0.12–11.81)	-
	Overweight	5.15(0.91–29.23)	3.43(0.74–16.03)	
	Obese	-	2.15(0.21–21.92)	
Waist circumference(cm)		1.06(0.99–1.14)	1.06(1.00–1.12)	**1.07(1.02–1.13)**
Abdominal obesity	Yes	**10.41(1.19–91.04)**	1.67(0.43–6.42)	1.79(0.58–5.54)
Physical exercise (hrs/wk)		1.03(1.00–1.06)	1.02(0.98–1.05)	0.99(0.94–1.05)
Smoking category (Reference = Non-smoker)	Ex-smoker		-	4.43(0.78–25.34)	**-**
	Current light smoker		0.98(0.11–9.04)	-	
	Current moderate smoker	**16.63(1.24–223.47)**	-		
	Current heavy smoker	-	**7.60(1.25–46.32)**		
Completed years of alcohol drinking		-	1.03(0.97–1.10)	**1.06(1.02–1.11)**
Type of alcohol drinking (Reference = Non-drinker)	Ex-drinker	-	5.61(0.55–56.97)	**22.43(4.62–108.81)**
	Current drinker	-	0.83(0.10–7.02)	1.66(0.33–8.43)
Family history of diabetes (Reference = No)	Yes	-	4.44(0.48–40.74)	1.11(0.27–4.62)
Diagnosis of hypertension	Pre-hypertensive	1.34(0.18–9.78)	0.67(0.06–7.56)	8.03(0.94–68.54)
	Stage I Hypertension	-	3.70(0.59–23.22)	7.41(0.74–73.88)
	Stage II Hypertension	4.39(0.58–33.27)	**6.58(1.02–42.33)**	**13.17(1.29–134.03)**

OR = Odds Ratio 95%CI = 95% confidence interval

Boldfaced figures indicate results for which p value was <0.05

“-”refers to situations when valid Odds Ratio could not be determined due to lack of adequate observations

Compared to laborers, those who are in services (OR = 43.00, 95%CI = 41.94–44.09) were more likely to have relatively better control, while business-holders (OR = 25.53, 95%CI = 24.91–26.18) and retired (OR = 46.53, 95%CI = 45.38–47.72) subjects were much more likely to have uncontrolled AGM. Subjects having abdominal obesity (OR = 10.41, 95%CI = 1.19–91.04) and current smokers (OR_Current moderate smokers_ = 16.63, 95%CI = 1.24–223.47; OR_Current heavy smokers_ = 7.60, 95%CI = 1.25–46.32) seemed to have better control over their diabetes while ex-smokers (OR = 4.75, 95%CI = 1.09–20.78), ex-drinkers (OR = 22.43, 95%CI = 4.62–108.81), those who were drinking for long (OR = 1.06, 95%CI = 1.02–1.11) and Stage II hypertensives (OR = 13.17, 95%CI = 1.29–134.03) had higher odds of having uncontrolled AGM. (Table 3)

## Discussion

In a representative sample of an urban adult population of West Bengal, the burden of AGM was found to be considerable. While FPG was found to be at impaired and diabetic level for 20.07% and 7.81% of individual s respectively, 26.02% had impaired and 9.67% had diabetic level of glycemic control. Based on the testing and standard diagnostic protocol,[18,19] 27.88% and 10.41% subjects were pre-diabetic and diabetic respectively. Including both previous diagnosis and test results during the study, the prevalence of diabetes was found to be 15.61% which was over 6% higher than the national level estimate of 9.01% by IDF for 2012.[6,11]Furthermore, among the previously diagnosed diabetes cases, 46.43% had uncontrolled AGM. These findings cumulatively indicated a large burden of AGM in the study population associated with known reversible factors. Therefore appropriately targeted intervention is necessary to reduce this burden.[35–38]

Subjects in this study were recruited randomly from an urban population cohort with a very low (7.24%) non-response rate. Hence considering the representativeness of the sample, the results revealed high prevalence of obesity, smoking, alcohol-drinking, hypertension, family history of diabetes and low mean hours of physical exercise among urban adults of West Bengal. As evidenced elsewhere,[13,35,39,40] the observed burden of lifestyle factors seemed to have the potential for increasing the risk of diabetes and its complications among the urban residents in West Bengal.

Corroborating with prior findings [11, 15, 30, 35–37, 40–43] increasing age was found associated with increased likelihood of being diabetic and of having uncontrolled AGM. Counseling by diabetic educators might minimize the problem by directing efforts to relatively older persons.

In this study, compared to laborers, retired subjects had higher odds (independent of age) of being diabetic while business-holders and retired were more likely to have uncontrolled AGM. Service-holders seemed to have relatively better control over aAGM. While advancing age, less physical activity and other co-morbidities were likely to increase the risk of diabetes among retired subjects lack, of social support, age-induced self-neglect and forgetfulness regarding medicines might be the explanation for poor control of AGM. While a relatively more routine life probably helped service-holders and house-wives to keep their AGM under control; stress, anxiety and instability probably increased the chances of uncontrolled AGM among business-personnel and unemployed subjects. Apart from few conflicting results,[30] prior evidences did also show that the unemployed, the retired and workers in higher income jobs were frequently found to be diabetic (and more often uncontrolled) compared to those with other jobs.[36,40,43].

Diabetic patients having stressful jobs, sedentary lifestyle along with those who are retired and unemployed should be targeted for better control.

Compared to those with normal body weight, overweight subjects in this study were more likely to be diabetic. Although obesity is an established risk factor for diabetes [11,37,40,44], there was no association between risk of diabetes with obesity in this study. This observation could be explained by the possibility that obesity, being a known risk factor, when compared to overweight subjects, obese diabetics might have been diagnosed and commenced on treatment earlier. Because of their knowledge regarding harmful effects of obesity, they could also have been more compliant with treatment. Consistent with this observation, a higher waist circumference was also associated with being diabetic and having uncontrolled AGM. A positive association of abdominal obesity with diabetes was also observed in prior studies.[11,15,36,37,39,40] Thus abdominal obesity appeared to be a stronger correlate of overall and uncontrolled AGM among urban residents in West Bengal. Hence screening for abdominal obesity should be included in all diabetic risk assessment tools considering this to be an important lesson from the study.

As in other studies [45–48] the current study showed that, compared to non-smokers, smokers were more likely to have diabetes [35] while current smokers had higher odds of having better control. Awareness of the harmful effects of smoking might have increased the compliance with control of AGM among smokers.

Compared to non-drinkers, ex-drinkers were also more likely to be diabetic while longer duration of alcohol drinking and being an ex-drinker were found to be associated with uncontrolled AGM. Previous studies also revealed a correlation between alcoholism and diabetes.[41] Reverse causation could be the explanation for this observation (i.e. people with alcoholism might have quitted drinking alcohol because of uncontrolled AGM) for both ex-smokers and ex-drinkers having higher chance of having uncontrolled AGM. Persons with a history of current or past smoking and alcoholism probably required special attention regarding control of AGM.

Subjects with a family history of diabetes also seemed to be at higher odds of having impaired FPG in this study. A genetic predisposition of AGM has also been reported from previous studies conducted in different states of India. [11,13,15,36,37,39,44,49]

The participants in this study had a high prevalence of hypertension as the proportions of subjects with pre-hypertension, stage-I hypertension and stage II hypertension were 33.09%, 17.84% and 12.64% respectively. A previous study in 2010, revealed a similar picture in West Bengal regarding the prevalence of hypertension (i.e. 46.5%).[41]Similar to previous studies,[11,15,39] in the current study, compared to normotensives, stage I and stage II hypertensives had higher odds of having AGM, while stage-II hypertensives were more likely to have uncontrolled AGM. Thus it is inferred that hypertensives need additional attention for good control of AGM.

This study has important limitations. Self-reported socio-demographic and behavioral information (e.g. smoking, drinking, exercise etc.) collected in this study had the potential to suffer from social desirability bias and the reporting of having previously been diagnosed as a diabetic might well have included some misclassification. Which also could be suspected as none of the female participants reported any history of smoking or alcohol drinking. However, as this study was nested in a long-term cohort, the trusted relationship between the research team and the participants could have helped in convincing the subjects adequately regarding the confidentiality of their information. Thus better recall and less chance of mis-reporting were likely to reduce the magnitudes of these information biases. Although we expect to have recruited a representative sample of an urban population of eastern India by virtue of our sampling design nested in a long-standing cohort, still the potential threat to generalizability owing to the emergence of new households in the study area over time and consequent missing of some eligible subjects should also be borne in mind.

Due to sample size issues (owing to lack of budget to conduct a larger study), some of the observations lacked power and multivariate analyses were not feasible. Thus we could only present the results of the bivariate analyses, but still consider them quite useful in developing important insight. However, while interpreting these results, the potential for confounding should always be borne in mind. Due to the cross-sectional nature of the study, causal interpretations of the results of the study were not possible. Temporal ambiguity is also an issue, although being nested in a population-based cohort, some amount of temporality was probably incorporated into any interpretation. Despite these limitations, by virtue of having a representative sample, nested in an urban cohort and having only 7% non-response, generalizability issues and selection biases were not likely to affect the study results.

## Conclusion

In this urban population of India, a high burden of total and uncontrolled abnormal glucose metabolism was observed, which was greater than the so-far reported prevalence in other communities of Indian subcontinent. Results suggested that waist circumference should be included as an important screening tool for diabetes.

Control of risk factors especially among relatively older subjects, retired, smokers, ex-drinkers and those having higher waist circumference is required to curb the diabetes epidemic in urban India. For harm reduction, appropriate counseling of the retired, business personnel, ex-smokers and ex-drinkers to ensure better control of AGM appears indicated.
